# One down, fifty to go: managers’ perceptions of their workload and how they cope with it to maintain their psychological health

**DOI:** 10.3389/fpsyg.2023.1336560

**Published:** 2024-02-05

**Authors:** Frédéric Boucher, Julie Dextras-Gauthier, Marie-Hélène Gilbert, Pierre-Sebastien Fournier, Justine Dima

**Affiliations:** ^1^Department of Management, Laval University, Quebec City, QC, Canada; ^2^Haute École d’Ingénierie et de Gestion du Canton de Vaud, Yverdon-les-Bains, Switzerland

**Keywords:** workload, managers (decision makers), coping strategies, psychological health, healthcare sector

## Abstract

**Background:**

Like many other countries, healthcare services in Canada face numerous organizational changes with the main objective of doing more with less. The approach taken within different healthcare networks has brought about a reform in healthcare facilities in Quebec, leading to several mergers and eliminating over 1,000 managerial positions. As a result, this has placed a progressively heavier workload on the shoulders of the remaining managers. Research on mental health in the workplace has mainly focused with the workforce and generally neglects managers. However, studies have shown that workload is a risk factor for managers. Therefore, the objectives of our study are to (1) better understand the elements that make up a manager’s workload and the factors that influence it and (2) identify the coping strategies used by managers to deal with their workloads.

**Methods:**

Employing a qualitative approach, we analyzed 61 semistructured interviews through an abductive method, utilizing diverse frameworks for data analysis. The participants came from the same Quebec healthcare establishment.

**Results:**

Our findings align with the notion that workload is a multifaceted phenomenon that warrants a holistic analysis. The workload mapping framework we propose for healthcare network managers enables pinpointing those factors that contribute to the burden of their workload. Ultimately, this workload can detrimentally impact the psychological wellbeing of employees.

**Conclusion:**

In conclusion, this study takes a comprehensive look at workload by using a holistic approach, enabling a more comprehensive understanding of this phenomenon. It also allows for the identification of coping strategies used by managers to deal with their workloads. Finally, our results can provide valuable guidance for the interventions aimed at addressing workload issues among healthcare network managers in Quebec by utilizing the specific elements we have identified.

## Introduction

Like many other industrialized nations, Canada is facing the issue of an aging population, new health technologies, and increased healthcare costs, which put pressure on its public healthcare system (Lyet and Molina, 2019). The demands for cost-effective health services are increasing, healthcare budget constraints are growing, and there is a shortage of clinical and administrative staff because of high employee absenteeism and turnover (Dubois et al., 2022). In this context, government officials, healthcare organizations, and their employees must constantly find new ways to reduce costs while improving the quality of services (Audenaert et al., 2019); in other words, they must find new ways “to do more with less” (Walker, 2014; Esteve et al., 2017). This situation generates continuous organizational changes and restructurings (Wilmar et al., 2017). For example, in the pursuit of cost reduction and centralization, the Quebec provincial government initiated a reform in 2015, reshaping the governance of the healthcare and social services network by creating centralized regional agencies known as integrated (university) health and social service centers (CIUSS and CISSS). The healthcare network reduced the number of establishments across the province from 182 to 34 (Gentile, 2016; Bourque and Lachapelle, 2018; Corbière et al., 2020). This merger led to the elimination of over 1,000 managerial positions (Gentile, 2016). More specifically, the number of managers has gradually fallen from 12,115 in 2012 to 9,327 in 2018. Despite this downsizing, managers’ administrative responsibilities have continued to increase (Ministére de la Santé et des Services sociaux [MSSS], 2019). This context had a direct impact on managers’ working conditions, resulting, among other things, in task transformations and increased job demands (Jung, 2014; de Jong et al., 2016; Corbière et al., 2020). Moreover, recent studies have indicated that the health and working conditions of healthcare managers have deteriorated, generating increased workloads, stress, and stressful working conditions (Wilmar et al., 2017; Corbière et al., 2020). These changing conditions affecting healthcare managers are associated with task fragmentation, growing uncertainties, conflicts of values, high-performance work pressures, hectic pace of work, long working hours, and turnover (e.g., Wilmar et al., 2017; Corbière et al., 2020; Gilbert et al., 2023). Managers find themselves overloaded, which has led to role ambiguity and decision-making challenges (Udod et al., 2017).

However, to properly play their leadership role, managers must be in a healthy work environment, they must be healthy themselves (Biron et al., 2018; St-Hilaire and Gilbert, 2019), and they must have favorable organizational conditions. To date, limited attention has been paid to the boundary conditions needed by managers to enact good management practices and leadership (Nielsen and Taris, 2019). These boundary conditions refer to the conditions experienced by managers, such as work demands and resources available, including their own individual resources (Nielsen and Taris, 2019). Considering the important role that managers play for employees (Parent-Lamarche and Biron, 2022), it is necessary to better understand the conditions in which the manager’s work is carried out daily. Corbière et al. (2020) found that healthcare leaders face numerous challenges related to the management framework, the management of human, financial, and material resources, the psychological health of their teams, collaboration, their employees’ sense of belonging, and work–life balance. In McConville and Holden (1999) highlighted the role dissonance of middle-line managers in the healthcare sector, even referring to it as “filling in the sandwich,” to illustrate their position between senior management and employees. Building on this, Korlén et al. (2017) identified the intermediary role that healthcare managers must play between the demands of employees and senior management. Considering these numerous roles, middle managers must assume their position in the hierarchy; they are facing conflicting demands from both employees and management that can have implications for their psychological health in the long term (Karanika-Murray et al., 2018). In this context, work overload is identified as a growing concern among middle managers in the healthcare sector (Bowling et al., 2015; Corbière et al., 2020), and it is recognized as a psychological risk factor influencing psychological health at work (Bakker and Demerouti, 2017). If the feeling of being overwhelmed by workload is shared by many managers and employees, efforts to understand this phenomenon suffer from a lack of consensus on the nature of workload, their antecedents, and consequences. Because the challenges to be overcome are numerous and the demand for healthcare services is growing, healthcare organizations need to be able to count on healthy and committed managers. Hence, it is necessary to gain a better understanding of managers’ working conditions and, more specifically, their workload. However, most studies on workload in healthcare settings have focused on nurses (Donnelly et al., 2016; Halpin et al., 2017; Fox-Robichaud et al., 2019; Johnston et al., 2019), medical specialists (Fox-Robichaud et al., 2019), and paramedical staff (Donnelly et al., 2016). However, the manager’s work has remained relatively absent from these studies (Fortin, 2014). Moreover, recent studies on the manager’s psychological health highlighted worrying issues and major constraints at work (Biron et al., 2018; Gilbert et al., 2019, 2023). Facing these constraints at work, individuals are known to use strategies to cope with deteriorated situations (Hobfoll, 1989). For example, nurses used rationing strategies to cope with heavy workloads, affecting the patient care provided (Van den Heede et al., 2009; Ross et al., 2019). In this context, studying managers’ workload implies considering work constraints as well as the strategies used to cope with them.

The objectives of our study are to (1) better understand the elements that make up a manager’s workload and factors that influence it and (2) identify the coping strategies used by managers to deal with this workload. By doing so, the present study makes three contributions to the field of psychological health. First, we address the gap in the literature by contributing to a better understanding of the manager’s work and workload, aspects that are forgotten in the literature (Fortin, 2014; Corbière et al., 2020). Second, our study focuses solely on healthcare network managers because the emphasis is often placed on other professions in the healthcare sector. Third, we focus on the strategies’ managers have put forward to cope with their workloads (Dellve et al., 2018). In doing so, our study considers the complexity of managers’ work activity in a dynamic environment, thus providing a better understanding of workload from a sustainable prevention perspective.

## Workload: a holistic-based approach

Traditionally, workload has been assessed from physical dimensions of work during a task (Laville, 2004) to individuals’ information processing capacity (Leplat, 1977). However, workload also covers a wide range of phenomena, adding to the complexity of defining and evaluating workloads (De Waard and Brookhuis, 1996). Matthews et al. (2020) noted three types of studies aimed at evaluating workload: (1) subjective assessment, (2) psychophysiological responses, and (3) performance measures. Although the subjective evaluation refers to the workload experienced, the psychophysiological reactions take into account the physical reactions (e.g., muscle contraction, ECG, EEG, etc.) and the performance measures used to evaluate the outcome of a task (e.g., errors, reaction time, quality, etc.). Matthews et al. (2020) considered these last two types of measurements to be objective because they are based on quantitative, measurable facts. However, it seems that they do not represent a direct measure of workload but rather a consequence associated with it. Moreover, these types of measurement are limited to a static analysis without considering fluctuations over time and according to situations or the subjective nature of the workload. As a result, most studies have failed to examine the workload phenomenon because it affects managers in their day-to-day work (Fantoni and Verkindt, 2015). Considering our objective of understanding the workload experienced at work, in the present study, we have adopted a subjective perspective.

Over the years, several instruments have been developed to evaluate subjective workload [e.g., the National Aeronautics and Space Administration – Task Load Index (NASA-TLX) (Hart and Staveland, 1988), the Subjective Workload Assessment Technique (SWAT) (Rubio et al., 2004), the Workload Profile (Rubio et al., 2004), etc.]. Even though these instruments point to important aspects, they provide little information on the complex and dynamic nature of workload (de Winter, 2014). To understand workload in a dynamic environment, we must approach it from the point of where a set of working conditions is taking shape in the daily activities of workers (Fournier et al., 2013). Understanding the workload experienced by workers requires a thorough understanding of their working environment and the conditions they face. Coron (2019) demonstrated that elements such as power relations, strategies, and leeway are the determining factors of perceived workload. Workload can then be defined “*as the intensity of effort provided by the worker to meet the demands of the task under determined material conditions, in relation to his or her condition and various mechanisms involved in his or her work*” (Teiger et al., 1973; Tort, 1974; Fournier et al., 2013, 48). Adopting this perspective brings us to a dynamic perspective of work activity (Daniellou et al., 2006; St-Vincent et al., 2011), where certain conditions and expectations generate a setting of working conditions. These conditions trigger specific activities, such as subjective perceptions, actions, and strategies by workers. This activity leads to consequences that affect performance, health, safety, leadership quality, and other factors, resulting in the transformation of existing working conditions. This dynamic perspective is closely linked to the situated action theory (Suchman, 1987), where human action is embedded in a specific context. Subjective workload is composed of three elements: prescribed workload, perceived workload, and actual (or real) workload (Falzon and Sauvagnac, 2004; Fournier et al., 2013).

1) ***Prescribed workload*** refers to the job demands and resources available to a person in their work context (Bakker and Demerouti, 2017). Job demands and resources can, for example, be performance expectations, work procedures, tools available, work constraints, work latitude, social support, and so forth (Nielsen et al., 2017). These demands and resources can also be found at the individual level, such as workers’ state of mind, experience, qualifications, health, fatigue, and so forth.
2) In this context of job demands and resources, ***perceived workload*** considers managers, subjective experience, and an assessment of the demands encountered in their job, including how they feel about the workload. For example, this perceived workload could be a feeling of being overwhelmed or being fulfilled by work demands while working (Fournier et al., 2013). This perceived workload then influences the actions deployed by the actor.
3) ***Actual (real) workload*** designates the activity deployed to achieve expectations in a specific work context (Fournier et al., 2013). This activity is composed of the actions and strategies developed by individuals or groups of persons to cope with the situations at hand (Jachens et al., 2018). In other words, when facing a specific situation involving demands and resources (prescribed workload), actors are experiencing different feelings (perceived workload) and deploying specific actions or strategies to face the situation at hand.

**FIGURE 1:**
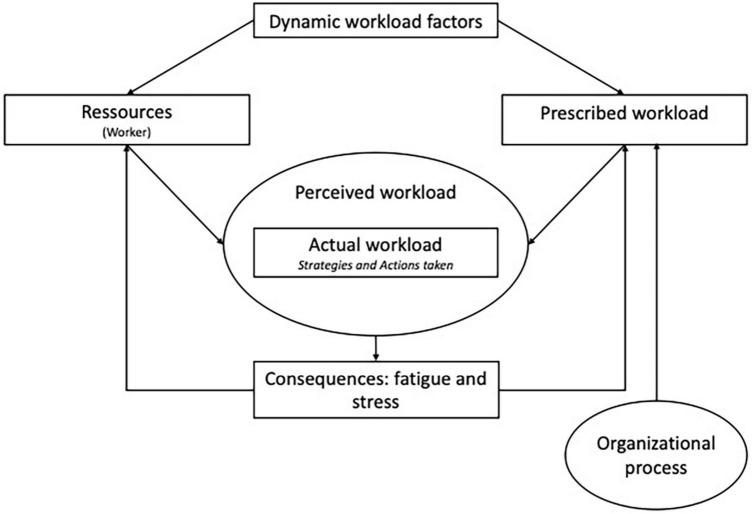
Fournier et al. (2013) model of subjective workload.

To fully understand the conditions under which work activity and, consequently, workload are built, we used the model proposed by Fournier et al. (2013) (Figure 1); this model considers workload to be situated in a specific dynamic organizational context, where actions are the result of an individual (or group of persons) with specific personal resources facing constraints and resources. These actions are situated and embedded in a perceived workload and strategies and generate various consequences (fatigue, stress, performance, quality, etc.). This situated perspective allows us to address subjective workload from a holistic point of view. In this model, *the prescribed workload* includes elements of the working environment such as the expected performance, the tools given by the organization to reach the expectations, and the procedures (De Montmollin, 1984). Moreover, prescribed workload is considered a constraint that evolves over time based on the directions taken by the organization (Fournier et al., 2013). In this context, *resources* refer to the experience, training, psychological and physical state of the person (Fournier et al., 2013). *Dynamic workload factors* are the larger organizational context that impacts the work setting of managers. *Perceived workload* corresponds to the subjective experience of facing a specific set of constraints and resources at work (Fournier et al., 2013). *Actual workloads* refer to the efforts made to achieve the prescribed workload (Fournier et al., 2013). Fournier et al. (2013) included in the *actual workload* all the work that could not be accomplished and remains to be done. *Consequences* correspond to the work activity while dealing with constraints and available resources. Finally, *organizational process* refers to the process that transforms the work conditions of the workforce. In the case at hand, the focus will be on the strategies and actions undertaken by managers to adapt to their workloads.

Upon the many possible actions and strategies that middle managers deploy in real work situations, the strategies used when facing overwhelming situations help in understanding the workload phenomenon. According to conservation of resources theory (Hobfoll, 1989), individuals use strategies to proactively deal with the loss of resources or when they must deal with diminished resources. The works of Hobfoll (1989) and Hobfoll et al. (2018) are also part of the theoretical foundations of job demands-resources (JD-R) theory, which suggests that job crafting is proactive work behaviors aimed at increasing resources and reducing job demands (Tims and Bakker, 2010). Some studies have reported that managers in public organizations used two major proactive strategies when confronting unhealthy working conditions: (1) accepting the situation by setting boundaries of time and energy investment into their managerial role or (2) distancing themselves from their managerial role and identity (e.g., Wilmar et al., 2017). To document the actual workload and strategies in context, we propose using the concept of coping strategies of Folkman and Lazarus (1980), which proposed two general types of coping. The first one—problem-focused coping—involves being in a state of problem-solving or acting with the goal of removing the source of stress. The second—emotion-focused coping—aims to reduce or manage the emotional distress associated with a situation. These actions and strategies deployed by managers will have effects on their own health (e.g., psychological health, fatigue), their ability to adopt positive leadership with their team, and eventually affect the health of their team members (Schaufeli and Enzmann, 1998; Skakon et al., 2010). Indeed, resources and constraints lead individuals to adapt their work to the conditions in which it is carried out. Hence, depending on the objectives, constraints, and resources at their disposal, people at work construct their work activity, which generates consequences (positive or negative) for the individual, group, and organization. This refers to the actual workload of the job, which encompasses operating methods, compromises, and strategies for overcoming work and organizational constraints (Lamonde and Montreuil, 1995; Fournier et al., 2013). There are several actions and strategies that individuals can deploy in their work process to cope with their workloads. In the present study, we focus on coping strategies as defined by Folkman and Lazarus (1980) because only a few studies have yet focused on this specific individual resource in relation to workload (Dellve et al., 2018).

Using this model allows us to document the elements that make up a manager’s workload and the factors that influence it. It proposes a dynamic perspective to identify the coping strategies used by managers to deal with their workloads. This approach differs from most studies seeking to measure workload in terms of overload, underload, or normal load, rather than seeking to understand the process itself. It also integrates the three elements of workload: prescribed workload, perceived workload, and actual workload.

## Materials and methods

### Design

To reach our research objectives, we opted for a qualitative approach, which allowed us to contribute to the literature by examining social interactions and individual experiences within the relevant environment (Denzin and Lincoln, 2008; O’Brien et al., 2014). We present the methodology following the structure outlined by O’Brien et al. (2014). The qualitative analysis of the interviews employs an abductive approach, as described by Timmermans and Tavory (2012), which involves an inferential and creative process of generating new hypotheses and theories based on research evidence. This approach facilitates theory development or refinement (Vila-Henninger et al., 2022). As pointed out by Vila-Henninger et al. (2022), qualitative abduction enhances the exchange between the literature and gathered data, fostering the development, expansion, and refinement of scientific knowledge. Abduction differs from both inductive and deductive approaches, but incorporates elements of both types of inference. This involves embedding emerging findings (induction) into existing theoretical models (deduction), thereby expanding their applicability to various contexts (Vila-Henninger et al., 2022). This aligns with Peirce (1974) suggestion of the iterative nature of analysis, encompassing induction, deduction, and abduction. Among the three approaches, abduction is the most speculative (Timmermans and Tavory, 2012).

### Participants and procedure

The present study is part of a larger research project conducted between 2016 and 2020 in a healthcare establishment in the province of Quebec, Canada. For the current paper, we use the qualitative data from the larger project. The data were collected from February to June 2017 using a purposive sampling method, which ensured the precise selection of sample items and adherence to the inclusion criteria established in the previous phases of the research project by the primary researchers (Royer and Zarlowski, 2014). The managers who participated in the first wave of quantitative data collection were invited to participate in individual interviews for the qualitative part of the study. The participation rate was 73% (*n* = 61 managers), and the study population included frontline, intermediate, and executive managers. The average age of the participants was 45 years (SD = 8.02), with 74% of them being women. Within our sample, 79% held clinical management positions, while 21% held administrative management positions. On average, the participants had between 6 and 10 years of management experience. The interviews were conducted exclusively in French because all the participants were fluent in the language. Participation was voluntary and anonymous, with participants signing a consent form prior to the interviews.

Given the exploratory nature of the research project, a semistructured interview was selected because of its flexibility (DeJonckheere and Vaughn, 2019). The interview was structured into distinct sections for a more organized discussion. In the initial section, the goal was to create a comfortable environment for participants, delve into their expectations, and clarify the objectives of the research project. The second section focused on the transition from a professional to managerial role. Two questions were presented: What factors contribute to your effectiveness in carrying out managerial duties? What factors pose challenges to your role as a manager? Finally, the fourth section of the interview delved into the managers’ perspectives on the impending cultural change, particularly concerning lean management. The approach we have adopted was inspired by activity analysis (St-Vincent et al., 2011) and was used to document workload in the day-to-day life of managers along three dimensions: prescribed workload, actual workload, and perceived workload (Fournier et al., 2013). In addition, this approach enabled us to document the dynamics that characterize managerial work and identify the determinants of managerial workload in terms of context, resources, and constraints. This approach also allowed us to identify the coping strategies implemented by managers to deal with their workload, as defined by Folkman and Lazarus (1980).

### Ethical considerations

The study protocol received approval from the research ethics committee of the participant healthcare facility. The participants provided written informed consent to participate and consented to the recording of the interviews. At the beginning of each interview, the interviewer reiterated that the interview would be recorded for the purpose of analysis and assured the participants that it would be anonymized to maintain confidentiality. Thus, implicit consent from the participants was obtained prior to starting the interviews. No compensation was given. To respect the anonymity of the participants, the excerpts will be presented with the participant’s code (e.g., P41) as well as the line where this excerpt can be found verbatim (e.g., P41: 98).

### Analysis

In the spirit of maintaining sensitivity to the research objectives in the first phase of data analysis, the code structure developed by the primary researchers was analyzed before beginning the secondary analyses. For the secondary analysis phase, all 61 interviews were coded a second time using MAXQDA 2022 software. This second analysis of the data was conducted with the goal of documenting the coping strategies that managers use to face their workload. The development of the codebook was based on the literature and data collected. Thus, Folkman and Lazarus (1980) model was used for the codes concerning coping strategies. Regarding the perception of workload, according to the definition proposed in the theoretical framework (Teiger et al., 1973; Tort, 1974; Fournier et al., 2013, 48), the elements perceived by the managers as being part of their workload were grouped together in Fournier et al. (2013) model. Codes were generated based on the subjective experiences of the participating managers (Blais and Martineau, 2006).

## Results

Based on our model, our analyses allowed us to conduct an examination of the workloads of the participating managers. Although workload perception was not explicitly addressed during the interviews, the managers referred to situations that they perceived as difficult in their work. Initially, the model we used allowed us to achieve our first objective, which was to better understand the elements that constitute a manager’s workload and the factors that influence it. Subsequently, we observed the coping strategies, as conceptualized by Folkman and Lazarus (1980), employed by the healthcare managers to deal with their workloads; this helped us achieve our second research objective. It must be kept in mind that what is perceived as a burden for one manager may not necessarily be the same for another.

### Contextualizing workload among healthcare managers

#### Prescribed workload

Our results showed various constraints characterizing the prescribed workload of managers in their daily lives. First, healthcare establishments such as hospitals operate 24/7 with teams working on shifts. On average, the participant managers supervised 52 employees working around the clock, and many frequently oversaw multiple work teams operating on three distinct schedules. To apply proximity management, managers must be present at key moments throughout the day and night, generating, among other things, schedule constraints: *“I’m there for all three shifts. I arrive before the end of the night shift and am there afterwards for the evening shift”* (P53: 58). Planning their arrivals and departures to see all their employees allowed them to be available for addressing emergencies, but it did not enable them to be consistently present and handle issues as they arose (e.g., during conflicts). Moreover, the adoption of proximity management practices was particularly valued in the establishment studied, putting a great deal of pressure on managers to implement this type of behavior without considering the various constraints experienced by managers.

Aside from team support, the managers performed multiple administrative duties, and to be effective, these duties often needed concentration time. *“For other managers, who have teams of 100–130 employees, making schedules and revalidating all that, […]. She can spend one day of her week doing that”* (P41: 98). Obtaining this focused time appeared to be more challenging for clinical managers compared with administrative managers. As the number of employees increased along with the complexity of situations, proximity management meant frequent interruptions and demands that represented a significant constraint for administrative duties and could put performance pressure on managers daily. “*It’s silly but managing a team and files or not managing a team and just managing files, you perform better*” (P35: 38). With the responsibility of managing people, there is also a significant volume of communications to handle. Communications constitute an important constraint. Instant messaging, emails, and other means of communication are challenging tasks and a source of stress among managers: *“If you boil it down to a normal week, I’d say I get between 50 and 75 (emails) a day”* (P30: 104).

Managers also served as the drop-off point for all demands from their employees and their organization. Organizational expectations are integrated into a culture of high-performance expectations: *“We have to perform; we have to meet expectations”* (P32: 65). Our interviews revealed that this performance culture was well integrated and shared by many participants: “*That’s the way things are done here”* (P32: 105). This organizational culture, as an organizational process, generated multiple constraints that affected the prescribed workload and, for instance, led managers to work beyond regular working hours. For example, mandatory meetings could add up on top of already busy schedules throughout the day and evening to accommodate time constraints: *This week, there isn’t a single evening where I am at home, and it’s not because I choose to catch up and work; these are unavoidable meetings that are part of the regular process of a director’s schedule* (P11: 12). Also, the nature of hospital settings meant frequent emergencies where prioritization was a challenge because everything can be considered urgent and needing to be addressed immediately. Furthermore, some managers worked in environments at the cutting edge of medical knowledge, so they also had to manage international competition in terms of scientific knowledge: *“Especially in an environment like ours, which is very competitive. We’re a tertiary center; we want so much to be the best;(…) we even want to be the best institute on the planet, and there’s a lot of emulation too, (…), we’re in the centers where excellence is made; we have a worldwide reputation here”* (P51: 136).

#### Resources

Our analyses identified the resources that empower managers to address situations and act. Primarily, managerial experience helped evaluate what can be achieved in each context. For example, having knowledge of organizational dynamics and processes helped negotiate which projects should be prioritized and which could be put on the sideline. In the following excerpt, a participating manager described the conversation he had with his superior regarding the implementation of various projects:

*I said, “Let’s put it all together. I can handle 20 projects, and I have a list of 150. We’ll draw a black line. These are the projects I’m going to work on […]. No one is going to work on these in the next 6 months.” They were all shocked. “But this one, we absolutely need to take care of it.” “Sure, give me a moment. You’ve given me resources, I’ve outlined how I’ll use them, I’ve analyzed, I’ve made a project list, and we even decided on prioritization criteria together, and I’ve prioritized them all. I’ve set an order, so don’t tell me I have to work on that one. I’ll get to it, but which one from the list should I remove?”* (P51: 144).

Experienced managers had a better knowledge of the environment and can also had less difficulty refusing requests: *“It’s been five and a half years since I’ve been here. I’ve become capable of saying, ‘No, this can wait.’ I now know what my current job entails”* (P38: 70). Additionally, one’s personality played a crucial role in managing organizational pressure: *“I believe that one’s personality and background contribute to one’s ability. I don’t intend that I’m better, but rather that your perspective on handling pressure is different” (P1: 58).* Hence, years of experience as a manager and one’s personality emerged as resources that can influence reactions to stressful situations, such as high workload.

#### Perceived workload

The perceived workload enabled us to gain an understanding of their subjective experience at work. The results showed that different life stages may influence how individuals perceived their workload and the importance of various factors, such as family or personal commitments: *“I believe I’m in the years where everything feels important, urgent, and demanding, and I wish I could allocate more time to everything all at once”* (P9: 148). This situation led some managers to feel overwhelmed because they were not able to complete everything asked and expected of them. “*At a certain point, you become overwhelmed because they keep adding new tasks to stimulate you, but at the same time, nothing is taken off your plate”* (P12: 144). When responsibilities keep adding up, the positive side of new challenges may not produce the expected positive results. In certain cases, they struggled to finish projects, which resulted in a feeling of not knowing where they were heading and a certain loss of sense in their work: *“What’s more, we cover several sectors, so requests come in from all sides. At first, you don’t know what you can say yes to and what you can say no to. (…). So you say yes to everything, and at some point, it just doesn’t make sense anymore”* (P38: 70). These overwhelming feelings brought managers to put aside everything that is not immediately essential: “*Let me put it this way. The workload makes it difficult to develop ourselves. You develop, but I’m registered for every webinar in the world, and I go to 1 in 5 because I never have time to do that. That’s a terrible obstacle*” (P33: 142). This subjective experience would influence the actions and strategies used by managers.

### Coping strategies used by healthcare managers

To date, we have provided an overview of the components concerning the prescribed workload, resources, and workload perceived by managers. Facing these constraints, managers need to find ways to cope with these situations. Our analysis was conducted based on the two categories of coping strategies identified by Folkman and Lazarus (1980): problem-focused coping and emotion-focused coping. Our analysis suggests that the managers relied more on problem-related coping strategies than on emotions.

#### Problem-focused coping

Problem-focused coping strategies refer to being in a state of problem-solving or acting with the goal of removing the source of stress (Folkman and Lazarus, 1980). The first strategy focused on prioritizing what one can do. The managers would previously convene on prioritization criteria and adapt these priorities to the situation while delegating what could be effectively done by another person: *“Time is elastic; I know my priorities; few jobs have truly got me worked up. It’s a matter of working on the right thing at the right moment. I made sure to delegate the rest to other people”* (P2: 214). Under pressure, this participant implemented a strategy to focus on urgent tasks while managing other responsibilities to make sure they were taken care of. Negotiating priorities and maintaining them to navigate through files and requests was a strategy that appeared to facilitate the maintenance of an efficient level of operation at work.

This led to another strategy: alone time management. This coping strategy involved allocating specific time periods in the schedule to tackle demanding tasks.

*I block off time to be able to work (…). When I’m focused, it is important to keep going, even if it means staying an hour later. It takes time to get going on a file and to be efficient; if you stop there and start again the next day, it is going to be hard, whereas if I’m in it, I’ll keep going. I’ve set myself some guidelines* (P35: 34).

First, this excerpt addressed the challenge of concentration and interruptions. Switching from one request to the other and then reimmersing oneself into the original task can be very demanding and inefficient. Maintaining concentration is a particularly significant concern for managers, especially given the substantial volume of incoming emails that constantly involve various requests directed at managers. These frequent interruptions have implications for both the performance and behavior of managers (Rosen et al., 2019). Second, alone time management strategies that could sometimes mean doing longer hours to complete a task—rather than having to resume work on a following day—can be seen as a relieving strategy. Our analysis revealed a strategy of overworking to achieve more projects with minimal interruptions. The managers exhibited heightened productivity beyond conventional work hours; they also raised the question of health consequences. We noted a few individuals who scheduled an additional 8 h of work at periodic intervals. Some managers pointed out that the absence of a team and interruptions allowed projects to progress at their maximum pace. Similarly, certain managers opted to take home some work and work on evenings or weekends to attain a similar sense of peace and quiet as those who remained at the office for extended periods: “*I try to bring home what I can bring home, and I tell myself, ‘Well, at least I’m doing it at home’*” (P18: 76).

On a similar topic, another problem-focused coping was communication management. Some strategies were used to limit solicitations, such as calls or text messages outside of normal working hours and maintaining some work/life balance. For example, managers would limit access to their cell phone number. Others would remove their office voicemail: *“I had my manager’s voicemail removed. It may sound trivial, but people were no longer capable of contacting me for everything. (…) if it is important, they’ll send an email”* (P26: 61).

Because email management was found to be a significant constraint, managers developed coping strategies to deal with them, such as in the evening or outside of their regular working hours:

*In general, (…) I coordinate everything even at home in the evening, which goes relatively well because I don’t elaborate in my emails, I put 2–3 lines, I can answer pretty quickly. I’m someone who, when he sees something elaborate, I call a meeting or tell him we’ll talk about it face-to-face because that’s quicker than email exchanges* (P56: 90).

Keeping responses concise and not elaborating seems to allow for more efficient management while being at home preventing email overload. *“I’ve made some drastic decisions like ‘I don’t work from home in the evenings anymore, except for emails*”’ (P11: 64). This manager separated email management from daily work-related tasks.

When team management provided psychological support, it could place an additional burden on the manager. This burden can be multiplied as the team increased and by situations encountered. One strategy was to refer to the resources available through the organization. However, the employees often preferred to confide in their manager; the strategy then consisted of being there for them: *“Even if there are resources. Employees often come to see you directly. (…) Even if you tell them there are human resources, and so on, the employees still come to see you”* (P28: 95). Although it is a sign of trust from employees, these situations added to the workload of managers as they become an additional responsibility on top of their existing duties. On the other hand, being there for their teams allowed them to solve issues in real time. For example, daily meetings every shift (night, day, and evening) can facilitate rapid interventions in developing situations. Finally, a problem-focused coping strategy is to skip breaks or lunch while working. In these situations, managers isolate themselves to catch up on their tasks and attempt to complete projects. One participant described this strategy as the “last resort” because it indicates that someone is not doing well: *“When you isolate yourself, when you don’t have time to go out to lunch; I don’t have time to go and see a colleague, to ask her what she would do, and you just get overwhelmed without taking the time to stop. The isolation of the manager, I believe, is the beginning of the end”* (P52: 42).

#### Emotion-focused coping

Emotion-focused coping strategies aim to reduce or manage the emotional distress associated with a situation (Folkman and Lazarus, 1980). To alleviate the stress stemming from requests, certain managers choose to tackle their tasks head-on rather than deferring them: *“But at some point, if I don’t do it, I end up with even more stress. So I prefer to take charge”* (P18: 78). For some managers, physical training was the most effective way to reduce workload-related stress outside of working hours. However, for some, it proved challenging to identify effective coping strategies for managing their workload and its associated consequences. Consequently, managers often resorted to a coping strategy that entailed taking time off from work: “*And I would say that it’s not… I’m trying to come up with strategies, and they are working relatively poorly, until the point where we must make drastic decisions like ‘getting someone to replace me’ or ‘not attending’*” (P11: 16). Just like the strategy of cutting down on break times, having someone fill in or take a leave of absence from work is the last-resort strategy employed by healthcare managers when the workload becomes unmanageable.

### Consequences for healthcare managers’ psychological health

Working in a demanding workload environment has consequences for healthcare managers. The first consequence that emerged in our analyses was stress. Although some coping strategies allowed managers to be efficient, it was mainly a response to this workload, aiming to alleviate the stress it imposed. In some cases, this stress persisted even when working outside of regular working hours: “*The fact that I’m still stressed because I can’t catch up on the weekend as quickly or get ahead*” (P33: 107). The second consequence was fatigue that could be felt mentally and emotionally: *“You go out at night, you’re more mentally tired, you’re more emotionally tired”* (P28: 95). Finally, for some managers, the workload became so heavy that they had to isolate themselves from their colleagues to try to meet all the demands. As this participant said, “*Isolation is the beginning of the end”* (P52). Managers who employed this strategy were not heading in the right direction in their efforts to reduce their workloads; they isolated themselves from the social support that could be provided by their colleagues.

### Subjective workload: a contextualized model

**FIGURE 2:**
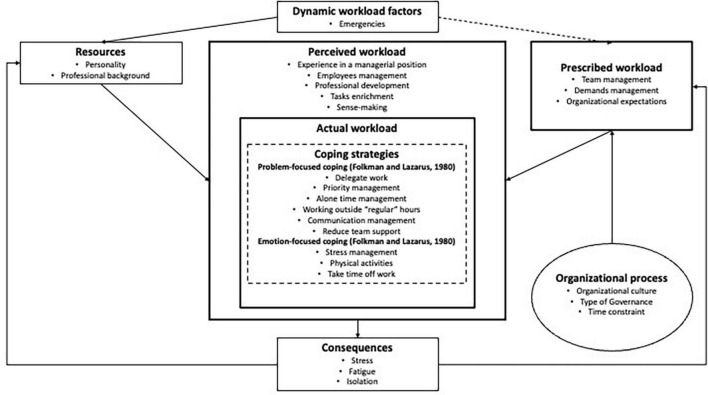
Managers in healthcare sector workload mapping.

In light of our results, Figure 2 shows the mapping of elements that comprised a manager’s workload and the factors influencing it, as proposed by Fournier et al. (2013). Every aspect of Fournier et al. (2013) model has been enriched with details specifically highlighted by the participating managers. These details are intricately tied to the unique context of the healthcare facility in which the managers operated. Figure 2 provides a portrait of the workload of managers contextualized to their healthcare institutions.

The central part of the figure comprising perceived workload, actual workload, and coping strategies delves into the subjective experience of managers. The manager’s perception of workload and implemented strategies played a crucial role in influencing their psychological health, as illustrated in Figure 2 under the consequence category. External elements encompassed the environmental factors influencing subjective workload, including components such as prescribed workload, dynamic workload factors, resources, and organizational processes. The arrow between dynamic workload factors was represented by a dashed line, indicating a lack of conclusive results to characterize this relationship. The results obtained led us to categorize the elements pertaining to the prescribed workload into three main areas: team management (size, schedule allocation, support, and conflict management); demand management (multiple projects, meetings, communication management); and organizational expectations (performance expectations and urgency associated with each request). Our results suggest that when combined, these three elements significantly increased the workload of managers. When we examine the perceived workload of healthcare managers—experience in a managerial position, employee management, professional development, task enrichment, and sense-making—the elements perceived as a burden do not necessarily pertain to the inherent nature of the work itself. Indeed, the significance of perceived workload and coping strategies employed by managers predominantly revolved around compensating for the time required to oversee work teams, address departmental directives (top-down directives), and manage emergent situations coming from dynamic workload factors. Regarding the actual workload, we have integrated coping strategies as suggested by Folkman and Lazarus (1980) and Fournier et al. (2013) model to offer a novel perspective on how managers in the healthcare sector coped with their work demands.

## Discussion

The objectives of the present article were, first, to gain a better understanding of the workload experienced by healthcare managers and, second, to explore the coping strategies they employed to deal with it in their daily work situations. A qualitative approach was employed to achieve our objectives, which involved 61 semistructured interviews. By acknowledging the multidimensional nature of workload, our study is in line with Hasson et al. (2023), who asserted that a unidimensional approach falls short of comprehensively addressing the entirety of the workload phenomenon. To achieve our first research objective, our model, inspired by Fournier et al. (2013), allowed us to assess managers’ working situations while detailing its components: dynamic context factors, resources, prescribed workload, perceived workload, actual workload, and the coping strategies that constitute it. The original model considered the actions and strategies within the actual workload in general without focusing on specific categories of strategy. We provide a contextualized perspective by focusing on the workload related to the work situations experienced by the participating managers. To reach our second research objective, we integrated the coping strategy concept (Folkman and Lazarus, 1980) in this perspective, imbedding actions to specific contexts (Suchman, 1987). By studying managers, we are also aligned with previous authors (e.g., Nielsen and Taris, 2019; St-Hilaire and Gilbert, 2019) who emphasized the importance of focusing on the psychological health of managers and pointed out that managers face unique challenges in their managerial roles, especially those related to their workload. We have also contributed to a better understanding of managers’ work and workload, addressing a previously identified gap in the literature (Fortin, 2014; Corbière et al., 2020). Our study also deepens the work of Gilbert et al. (2023), who highlighted the importance of managers’ workload as a main constraint encountered by healthcare managers that may affect their psychological health. In our analysis, we noticed those elements previously discussed by Jung (2014) and Corbière et al. (2020) concerning the intensification of workload in recent years. Furthermore, we have identified the factors related to stress stemming from working conditions, including high-performance work pressure, extended working hours, and a fast-paced work environment. We also noticed that, according to the position held by managers, they often found themselves in a “sandwich” situation (McConville and Holden, 1999; Korlén et al., 2017), where they must make decisions based on ministry demands, despite being fully aware that these demands are unachievable because of the on-the-ground context (Wilmar et al., 2017; St-Hilaire and Gilbert, 2019; Corbière et al., 2020). Through the representation of this workload mapping (Figure 2), we can observe that intervening in workload requires a more holistic understanding of the concept in the context of the intervention, involving prescribed workload, perceived workload, and actual (or real) workload.

The workload perceived by the managers seemed to be quite burdensome. The factors characterizing their workload, as illustrated in Figure 2, indicate a combination of various contributing factors that go beyond individual factors. Upon examining the coping strategies (problem-focused coping and emotion-focused coping), as defined by Folkman and Lazarus (1980), they are more problem (e.g., prioritizing) than emotion focused (e.g., physical training). Also, it is noticeable that some strategies used may have positive impacts (e.g., alone time management), but some strategies have positive short-term impacts (e.g., reducing stress) but are likely to lead to negative effects in the long run (e.g., bringing work home). In other words, the adaptive outcomes of these strategies can be considered both functional and dysfunctional (Bruchon-Schweitzer, 2002) and could generate additional workload. When we consider the strategy related to reducing direct support for teams, it may be functional for the manager because it conserves their energy and focuses on their tasks. It can also be dysfunctional for employees who lack support. Another example pertains to the coping strategies employed for email management. Interruptions associated with emails have been increasingly acknowledged as a significant source of stress for managers, which, in turn, has a detrimental impact on their managerial behaviors (Hertz, 2014; Rosen et al., 2019). Therefore, the need for effective adaptation mechanisms is paramount given the widespread use of email as a communication channel. Further studies will need to be conducted to understand the effects of the adaptation strategies used by managers on their employees. However, it becomes important to ensure that the “choices” of coping strategies do not hinder the completion of managerial work, especially when managers are overwhelmed. In other words, the demands placed on managers’ shoulders should not prevent them from fulfilling their managerial duties, which can be broadly categorized into two main categories: task direction and psychological support. Tafvelin et al. (2023) reported that a higher workload was significantly associated with more passive/cowardly leadership behaviors. In our study, we found that the components of leadership responsibilities such as task direction and psychological support, as proposed by Katz and Kahn (1978) and Lanaj and Jennings (2020), contributed to the perceived heaviness of the manager’s workload. Certain coping strategies are implemented by managers to mitigate the effects of the role-induced burden: working outside regular hours when it is quieter, managing communication channels to reduce volume, and scheduling to block off time to avoid disturbances. It becomes legitimate to question whether the responsibilities associated with a leadership role constitute the antecedents of workload.

Our findings have prompted us to explore the diverse ways managers cope. In the context of work overload, it appears that the managers predominantly reacted to this stressor by attempting to address the root causes of the problem and the associated stress. Hence, we could consider the coping strategies of Folkman and Lazarus (1980) as reactive strategies, given that managers use these strategies in response to workload as a stressor. Among the literature on proactive strategies, one may wonder if the job crafting concept proposed by Wrzesniewski and Dutton (2001) and by Tims et al. (2012) could apply to our managers’ workload context. According to Tims et al. (2013), employees tend to craft their tasks to enrich their work situations. Job crafting suggests four dimensions: (1) increasing structural job resources; (2) increasing social job resources; (3) increasing challenging job demands; and (4) reducing hindering job demands (Tims et al., 2012). Regarding the first dimension, our results (Figure 2) have indicated that our managers do not have the time to develop new resources, such as developing new skills, adding diverse tasks, or requesting training. The social job resource dimension has been identified as a key factor, particularly in terms of addressing isolation, which often leads to higher levels of psychological distress. Although some strategies (e.g., skipping breaks) may lead to isolation, this dimension appears particularly relevant for our managers standing to benefit from increasing their social resources (e.g., support of their own manager), as emphasized by Gilbert et al. (2023). The third dimension—increasing challenging job demands—does not seem suitable for the context of our managers, where responsibilities were frequently added to their workload, and at a certain point, managers lost work meaning. Adding tasks, even though stimulating, would also be considered additional responsibilities. Finally, reducing hindering job demands appears to be a major concern for these managers, especially for task delegation and improvements in communication management. Overall, job crafting seems relevant because it aimed to increase resources and reduce constraints, but it appeared to be more suitable for employees seeking to enrich their work compared with those trying to survive in an overloaded work context, as was the case with the managers in the present study. Reactive coping strategies appeared to better describe the actions taken by our managers who had to deal with stressors, such as workload.

### Practical contributions

By specifically focusing on the factors that impact the workload of healthcare managers, we can pinpoint specific intervention strategies that could yield positive outcomes. The contextual aspect necessitates engaging in discussions with managers about their work situations to better intervene in their environment and achieve a tangible impact. To effectively address workloads, it is crucial to consider various aspects of their work, fostering open dialog, and, thereby, developing a context-oriented approach for primary prevention. Intervening on resources appears to be a way to impact all three components of workload (prescribed workload, perceived workload, and actual workload). Indeed, healthcare organizations could improve managers’ workload through interventions aimed at building workplace resources at multiple levels in the organization. Following the IGLO model developed by Nielsen et al. (2017), organizations can improve resources at four levels: individual, group, leaders, and organization. At the organizational level, our results have shown that the organizational culture of the healthcare establishment studied seems to be one of performance. The question arises regarding the suitability of this type of culture within the healthcare environment because they are under public administration. The literature has demonstrated that there are conflicting values in public management, including a debate between efficiency and democratic legitimacy (Oldenhof et al., 2014). In our study, the values of performance and excellence, coupled with the “do more with less” approach (Esteve et al., 2017), gave rise to discrepancies between the expected tasks and achievable outcomes. The perception of these disparities also contributed to the workload of managers. This is especially evident in team management, where managers make requests fully aware of resource constraints. In a recent study on healthcare managers, Dextras-Gauthier et al. (2023) found that managers in a work environment that is characterized by a group culture putting a strong emphasis on human resources and promoting a supportive and healthy work environment reported fewer levels of psychological distress and higher levels of wellbeing. For healthcare organizations, questioning their organizational culture and the consequences associated with this type of culture on managers’ workload could be the first step toward creating healthier and safer working conditions based on a group culture (Dextras-Gauthier et al., 2023). Providing managers with job control and decision latitude could also be an important strategy for managers to better manage their workloads. Indeed, this could allow managers to choose when and how to delegate part of their workload to their employees or colleagues. At the leader level, managers themselves need the support of their superiors. Leaders (manager’s immediate superiors) are one resource to counter the negative effects of workload (Gilbert et al., 2023). Even though they are in a more competitive culture, managers consider their immediate superior to be a valuable ally and important resource for reducing their stress (Gilbert et al., 2023). Whether it is through advice, coaching, or just to feel understood, leaders are a significant resource for managers (Gilbert et al., 2023). At the group level, colleagues are an important resource for managers. Our results show that experience in a management position is a resource for prioritizing files, which helps in better managing the workload. Considering that management experience seems to help, organizations could set up a mentoring system in which a manager with little experience in a management position could be assisted by a more experienced one. Also, shared managerial assignments could also be a working condition that supports managers in better performing complex daily work practices (Dellve and Eriksson, 2017). This is all the truer because the managerial role is often perceived as a lonely one (Dellve and Eriksson, 2017). Providing more opportunities for managers to support each other in their daily work tasks could be one strategy for them to better manage their workloads. At the individual level, managers need to develop handling strategies for workloads and recovery. Setting boundaries of time and energy investments in their managerial role could be one strategy used by managers. A better understanding of their managerial role could also help with the management of their workload, but also to give room for recovering activities. Although overloaded managers tend to adopt more reactive coping strategies, they still benefit from adopting proactive strategies, as suggested by job crafting (e.g., seeking help, pursuing training). These strategies could facilitate the acquisition of resources that may help them gain more control over their workloads. Employees are also an important part of managers’ work activities (Kurke and Aldrich, 1983). Employees interact and develop unique relationships with their leaders (Graen and Uhl-Bien, 1995). The attitudes and behaviors of employees can positively or negatively influence the workload of managers. For example, managing less autonomous employees with attitude problems could increase managers’ workload and stress (Gilbert et al., 2023). Helping employees who struggle emotional problems could also lead to role overload for managers (Bolino et al., 2015) and have detrimental consequences for them, such as citizenship fatigue and work–family conflict (Toegel et al., 2013; Lanaj and Jennings, 2020). Having too many employees to supervise could also negatively impact managers’ workload (Wallin et al., 2014). St-Hilaire et al. (2019) highlighted the role of employees in creating a good work environment for their leaders. By getting the work done, keeping the manager updated on team issues, taking the initiative, showing concern for their manager, and being honest and open, employees contribute to creating a healthy work environment for their managers, which shows the importance of raising awareness among them on this subject (St-Hilaire et al., 2019).

Our findings have provided valuable insights into managers’ experiences concerning their workload perceptions. Given the crucial nature of this issue for contemporary managers, our results not only validate the experiences of others, but also help individuals pinpoint the factors contributing to their own perceived workload. Analyzing the workload phenomenon allows for the identification of multiple intervention opportunities, whether related to organizational culture, assigned workloads, coping strategies, or the resulting consequences. As a result, organizations can implement more precise interventions, and employees can take proactive steps in areas they can influence, such as the strategies they employ and outcomes they achieve. Finally, intervening in workload necessitates considering multiple factors to effectively instigate substantial changes. Nonetheless, by intervening at the source of an element perceived to contribute to the burden of workload, the effects could manifest across different levels. This is even more important because those managers exposed to higher workloads are less likely to engage in transformational and transactional leadership behaviors and more likely to engage in laissez-faire leadership behaviors (Rosen et al., 2019; Sherf et al., 2019). In a context where managers need to combine multiple roles and tasks (administrative, instructional, and informational roles), as in the healthcare sector, higher workloads can quickly become overwhelming and impede managers’ ability to successfully engage in all aspects of their work (Bakker and Demerouti, 2017; Tóth-Király et al., 2023). Helping managers achieve more acceptable workloads could, in turn, help healthcare organizations attract and retain managers.

### Limits and avenues for future research

Given the exploratory nature of the present research project, there are several limitations that should be acknowledged. Although workload challenges resonate with managers in general [e.g., St-Hilaire and Gilbert (2019), the data collection was limited to Quebec and one healthcare facility, making it pertinent to replicate the study not only within diverse health organizations and sectors, but also among managers from different provinces and countries]. The composition of our sample leaned heavily toward clinical managers (79%) in contrast to administrative managers (21%), which could have influenced the outcomes reflecting the realities of their daily lives. Exploring the variations in workload perceptions between these two groups can offer valuable insights. Furthermore, the study’s sample predominantly consisted of women (74%), aligning with the gender distribution in the healthcare system. Examining gender distinctions could enable us to identify whether coping strategies are more frequently employed by men or women and to evaluate their adaptive effects based on gender. Employing a quantitative design would be suitable for conducting this type of study. A diary study could also be interesting because it would enable researchers to track the day-to-day workload that managers face. It is important to note that the interviews were conducted with managers from a healthcare facility within the Quebec healthcare system. The results may be shaped by the prevailing culture within this organization and broader cultural context of the province of Quebec. Therefore, any interpretation should carefully consider the factors at play. Replicating the current study in a different context has the potential to enhance our understanding of the workload experienced by managers. Finally, given the constrained time frame of the semistructured interviews, certain subjects pertaining to the study’s objective may not have been explored in as much depth as desired.

## Conclusion

The present study helped us gain a better understanding of the workload complexity faced every day by healthcare managers. More specifically, we were able to identify elements contributing to their workload using Fournier et al. (2013) model and integrating coping strategies as conceptualized by Folkman and Lazarus (1980). Workload is a particularly complex phenomenon, and its study requires looking at the top from various perspectives. Healthcare sector managers are known to carry a significant workload, making them a particularly rich sample for understanding their perceptions, experiences, and coping strategies. The present study also made it possible to identify the various coping strategies used by managers to handle their workload. These strategies appear to be more reactive than proactive. The current study opens the door to identifying possible interventions at different levels, involving the organization, the managers themselves, their superiors, their colleagues, and their employees. In a context where a new reform is looming in the Quebec healthcare sector, it is crucial to give more consideration to managers and their substantial workloads. This is especially important given their key role in organizations, not only for retaining healthcare personnel who struggle to stay in their roles as our society grapples with a major labor shortage, but also for ensuring the quality of care in an aging population. In addition, the role of a manager is losing popularity, so it becomes crucial to enhance the attractiveness of this position.

## Data availability statement

The data analyzed in this study is subject to the following licenses/restrictions: access to the data is restricted to protect confidential information. The data could be available upon request with permission of the healthcare facility and participants. Requests to access these datasets should be directed to JD-G, julie.dextras-gauthier@fsa.ulaval.ca.

## Ethics statement

The studies involving humans were approved by Comité D’éthique de la Recherche de l’Université Laval and the study protocol was approved by the participant healthcare facility’s research Ethics Committee. The studies were conducted in accordance with the local legislation and institutional requirements. The participants provided their written informed consent to participate in this study.

## Author contributions

FB: Conceptualization, Formal analysis, Methodology, Writing – original draft, Writing – review and editing. JD-G: Funding acquisition, Resources, Supervision, Writing – review and editing, Data curation, Writing – original draft. M-HG: Supervision, Writing – original draft, Writing – review and editing, Data curation. P-SF: Writing – review and editing. JD: Formal analysis, Writing – review and editing, Data curation.
